# Editorial: Public health policy and health communication challenges in the COVID-19 pandemic and infodemic

**DOI:** 10.3389/fpubh.2023.1251503

**Published:** 2023-08-04

**Authors:** Zhiwen Hu, Chuhan Wu, Pier Luigi Sacco

**Affiliations:** ^1^School of Computer Science and Technology, Zhejiang Gongshang University, Hangzhou, China; ^2^Collaborative Innovation Center of Computational Social Science, Zhejiang Gongshang University, Hangzhou, China; ^3^Department of Electronic Engineering, Tsinghua University, Beijing, China; ^4^Department of Philosophical, Pedagogical and Economic-Quantitative Sciences, G. d'Annunzio University of Chieti-Pescara, Pescara, Italy; ^5^metaLAB (at) Harvard, Cambridge, MA, United States; ^6^Institute for the Sciences of Cultural Heritage, National Research Council, Naples, Italy

**Keywords:** public health policy, health communication, COVID-19 pandemic, COVID-19 infodemic, large generative models

## Introduction

On 2 February 2020, the World Health Organization (WHO) characterized the COVID-19 infodemic as an overabundance of information, “some accurate and some not—that makes it hard for people to find trustworthy sources and reliable guidance when they need it.” Indeed, this assessment sheds light on the fact that we have struggled with both the COVID-19 pandemic and co-evolving infodemics (e.g., disinformation, misinformation, fake news, rumors, and lies) in the aftermath of the COVID-19 pandemic, as well as the need to foster interdisciplinary collaborations to fill crucial niches in public health policy and health communication (1–7).

The COVID-19 pandemic is fueling digital health transformation, accelerating innovations of digital health services, surveillance, and interventions, while further amplifying the social impact of deliberate COVID-19-related disinformation and misinformation activities. However, there is a relatively limited amount of research worldwide that has focused on the advancements in digital health innovations and surveillance strategies in the crux of both the COVID-19 pandemic and the COVID-19 infodemic from multidisciplinary perspectives, including proven innovations in public policy evaluation (PPE) (8).

The Research Topic “*Public health policy and health communication challenges in the COVID-19 pandemic and infodemic*” includes 14 articles reporting on research findings regarding public policy evaluation (PPE) with five overarching themes, including nine original research studies, two brief researcher reports, two reviews, and one perspective. The foci of these articles, published in the Frontiers journals *Frontiers in Public Health, Frontiers in Medicine*, and *Frontiers in Education*, are diverse, broadly including:

- Innovative approaches to public policy evaluation (Liu and Jiang; Carr et al.; Li et al.; Mejia et al.; Xu et al.).
- Public perception and collective behaviors (Wibowo et al.; Xue et al.; Carr et al.; Nagarajan et al.; Lee et al.; Gerretsen et al.).
- Innovative communication strategies against the COVID-19 infodemic (Adhikari et al.; Lee et al.; Hu et al.).
- SARS-CoV-2 vaccine inequity and vaccine hesitancy (Chen et al.; Shobako; Hu et al.).
- The challenges of science-based policymaking (Li et al.; Lee et al.; Wibowo et al.).

## Think globally, act locally

The burdens of the COVID-19 crisis span both the direct health and societal impacts of the virus as well as the indirect impacts from the accompanying information environment. An infodemic response that promotes an accurate and consistent science-based narrative, while also supporting public mental health and wellbeing, is needed alongside measures to curb the actual spread of the virus. The crux of the issue is that we must control both the COVID-19 pandemic and the COVID-19 infodemic to overcome this global crisis. Failure in either domain will undermine the progress made in the other. An effective response requires international cooperation on both fronts.

Controlling the pandemic and infodemic requires global cooperation using place-based, tailored strategies because standard policies and messaging will not suit all social context and needs. Public health depends on addressing both the disease spread and the spread of accurate information that resonates with diverse experiences. In this Research Topic, researchers offer simple but compelling recommendations that encourage people to start making a difference in their community on issues that matter globally.

Evidence shows compliance with recommended measures depends on more than rules alone. It relies on a mix of factors like beliefs, traits, needs, and mental health that differ by groups. Alternative interventions may be needed to motivate change when experiences do not. Studies also found disproportionate impacts, needs, and information use in diverse populations based on gender, culture, vulnerability, and more. For example, Gerretsen et al. found adherence to social distancing during the COVID-19 pandemic depended on a mix of demographic factors, beliefs about the virus, personality traits, psychological needs, and more in the U.S. and Canada. While adherence was generally good, influencing the factors within our control, like risk perceptions and social support, can help strengthen public resolve, especially in the long term.

Research from across India, Latin America, Indonesia, and elsewhere shows success where policies and information were adapted to local contexts, barriers, and groups, and failure where not. Messaging must reach the vulnerable. Policies and technology improve responses, but depend on equity, inclusion, and understanding differences. In a cross-sectional study, Adhikari et al. examined the factors associated with holding stigmatizing views toward infected people and experiencing stigma as a recovered patient during India's first COVID-19 wave. Significant levels of stigma were found in communities and reported by recovered participants. Several sociodemographic factors were linked to higher stigma. Nagarajan et al. found adults in Chennai, India, generally knew masks reduce COVID-19 transmission, but many remained opposed to mask mandates. However, mask wearing when outside was still common. Knowledge was lower and attitudes less favorable in slum populations. Mejia et al. found education level and country of residence were associated with basic COVID-19 knowledge in Latin America. Most had knowledge of symptoms and transmission, but gaps remained in some areas. Peru's low knowledge and high case rates suggested limited health literacy may worsen outbreaks. Wibowo et al. found belief in health consequences motivated uptake of COVID-19 prevention behaviors during Ramadan in Indonesia, but psychological, social, and resource barriers also undermined adherence for some. Promoting new behaviors depends on recognizing their impacts in context.

Policies in the UK, Japan, and Australia aimed to curb infection but may have negatively and disproportionately impacted children and women. Balanced, evidence-based policies also consider wellbeing, development, and mental health. Information use depends on more than just access to facts. Anxiety and care duties shape experiences. In a brief research report, Carr et al. examined whether the COVID-19 pandemic impacted people's disgust sensitivity in UK adults, especially toward pathogens and COVID-19. The results found that both overall disgust sensitivity and COVID-19-related disgust sensitivity remained unchanged, despite the significant life disruptions and health crisis experiences during the pandemic. This suggests disgust sensitivity is stable and current experiences alone may not be enough to motivate behavioral changes during infection prevention and control (IPC) measures. The implications are that alternative interventions, possibly leveraging disgust, could still be useful for promoting compliance with recommended COVID-19 measures. Hu et al. argued that the disproportionate impacts of COVID-19 on South Asians in Britain reflected systemic racism that must be addressed for an equitable, just society. While vaccine hesitancy and health inequities were symptoms, the root causes ran much deeper. Tackling racism requires education, and decolonizing the secondary curriculum to teach cultural awareness, promote inclusion, and build understanding is key. In a perspective paper, Shobako et al. argued that Japan's health policies for COVID-19, while aiming to protect public health, may disproportionately and negatively impact children. The policies disrupt school, diet, physical activity, and development. They are also often promoted more by public opinion than by evidence. The article calls for policies that are balanced, evidence-based, protective of children's wellbeing, and informed by diverse experts and feedback. Health and development must be considered alongside just infection control. Lee et al. found Australians used authoritative sources for urgent COVID-19 information to enable decision making and daily activities. Some changes occurred in favor of better accuracy and timeliness. But anxiety and disproportionate mental burdens, especially for women managing care duties, require consideration in strategic response. Their experiences highlight that information use depends on more than access or proximity to facts alone.

In China, Liu and Jiang examined factors influencing individuals' compliance with the Chinese government's COVID-19 preventive measures during regular prevention and control. The results showed that greater media exposure significantly predicts higher perceived severity, maladaptive rewards, self-efficacy, response efficacy, and response cost. Perceived severity, self-efficacy, and response efficacy positively predict protection motivation, which predicts compliance. Protection motivation also positively affects compliance through implementation intention. Perceived cultural tightness–looseness moderates the effect of protection motivation on implementation intentions, such that the effect is stronger with higher perceived tightness. Xue et al. found most people in China reported following recommended COVID-19 preventive behaviors, but information sources influencing behaviors differed in various groups. While internet resources had the largest impact overall, more tailored guidance through family doctors and community health centers was important for more vulnerable populations. In a critical review by Chen et al., the modeling study compared how COVID-19 and influenza might spread in a hypothetical city under different scenarios in China. They found that vaccination has greater potential than non-pharmaceutical interventions (NPIs) alone for curbing influenza, while a combination of emerging COVID-19 vaccines and NPIs will likely be needed to control surges. But vaccination can transition societies to less restrictive, sustainable measures if caseloads are reduced sufficiently over time. Xu et al. analyzed the Omicron subvariant BA.5 outbreak and response in Macau and found that while highly transmissible, the subvariant could be effectively contained through a multi-pronged strategy. Coordinating vaccination, social measures, testing, tracing, and treatment helped curb the spread. Despite its high population density, Macau achieved a lower infection rate than other regions facing BA.5. An integrated policy including the innovative “relatively static” plan was key.

Furthermore, AI and modeling require diverse, updated data to improve performance and match changes. In an AI-powered assessment, Li et al. introduced a multistage multimodal deep learning (MMDL) model that uses consecutive rounds of symptoms, test results, and other data to determine COVID-19 severity and predict worsening conditions in Chinese patients. The proposed approach outperformed single-point or single-modal models. However, more diverse, larger datasets—especially for severe patients—are needed to improve performance. The model must be re-tested and retrained to keep up with viral changes. If validated, this approach could help identify high-risk patients early for treatment.

## Peril and promise

In this Research Topic, the research makes a persuasive case for coordinated but locally-adapted strategies to address multifaceted global crises like the COVID-19 pandemic and infodemic. A one-size-fits-all approach will fail; progress depends on addressing diverse populations based on understanding differences in experiences, needs, and obstacles. Studies worldwide show why equity, inclusion, and place-based interventions matter. Public health success requires integrated, tailored strategies fitting local contexts. Outcomes depend on tailored solutions for populations, not policies serving assumptions. They rely on grasping various realities and motivating change by building knowledge and enabling action from within. Broad policies risk overlooking marginalized groups; targeted support and education are required to overcome barriers, curb disease, and combat informational threats straining social cohesion. Culturally-sensitive, anti-racist strategies can promote inclusion when crises test communities.

Though the WHO canceled the PHEIC (Public Health Emergency of International Concern) statuses for COVID-19 and Mpox in May, the threat remains. The rising concern now is that emerging large generative models (LGMs) like chatbots may proliferate future infodemics by generating false guidance or impersonating people online at a speed and scale overwhelming official information and responses (9–11). Without mechanisms ensuring transparency, oversight, and precision, chatbots like ChatGPT could spread infodemics quickly, fueling confusion and hampering crisis response (12). With planning and prudent policies, these technologies can support response; without them, they imperil it.

While such issues seem overwhelming in scale and scope, progress starts small, through raising awareness, personal action, community involvement, and advocating local policy changes (13). Together, these steps drive real change. But it begins with a global mindset and local solutions.

## Author contributions

ZH reviewed the literature and wrote the editorial. CW and PS reviewed the literature and edited the editorial. All authors read and approved the final editorial.
